# Mimosa Kinetic Façade: Bio-Inspired Ventilation Leveraging the Mimosa Pudica Mechanism for Enhanced Indoor Air Quality

**DOI:** 10.3390/biomimetics8080603

**Published:** 2023-12-13

**Authors:** Sukhum Sankaewthong, Kazunori Miyata, Teerayut Horanont, Haoran Xie, Jessada Karnjana

**Affiliations:** 1 Japan Advanced Institute of Science and Technology, Nomi 923-1211, Ishikawa, Japan; xie@jaist.ac.jp; 2 School of Information, Computer and Communication Technology, Sirindhorn International Institute of Technology, Thammasat University, Bangkok 10200, Pathumthani, Thailand; teerayut.horanont@gmail.com; 3 National Electronics and Computer Technology Centre (NECTEC), Bangkok 10400, Pathumthani, Thailand; jessada.karnjana@nectec.or.th

**Keywords:** kinetic façade, biomimicry, air ventilation, *Mimosa pudica*, air change rate

## Abstract

In light of pressing global health concerns, the significance of indoor air quality in densely populated structures has been emphasized. This research introduces the Mimosa kinetic façade, an innovative design inspired by the adaptive responsiveness of the Mimosa plant to environmental stimuli. Traditional static architectural façades often hinder natural ventilation, leading to diminished air quality with potential health and cognitive repercussions. The Mimosa kinetic façade addresses these challenges by enhancing effective airflow and facilitating the removal of airborne contaminants. This study evaluates the façade’s impact on quality of life and its aesthetic contribution to architectural beauty, utilizing the biomimicry design spiral for a nature-inspired approach. Computational simulations and physical tests were conducted to assess the ventilation capacities of various façade systems, with a particular focus on settings in Bangkok, Thailand. The study revealed that kinetic façades, especially certain patterns, provided superior ventilation compared to static ones. Some patterns prioritized ventilation, while others optimized human comfort during extended stays. Notably, the most effective patterns of the kinetic façade inspired by the Mimosa demonstrated a high air velocity reaching up to 12 m/s, in contrast to the peak of 2.50 m/s in single-sided façades (traditional façades). This highlights the kinetic façade’s potential to rapidly expel airborne particles from indoor spaces, outperforming traditional façades. The findings underscore the potential of specific kinetic façade patterns in enhancing indoor air quality and human comfort, indicating a promising future for kinetic façades in architectural design. This study aims to achieve an optimal balance between indoor air quality and human comfort, although challenges remain in perfecting this equilibrium.

## 1. Introduction

In the wake of recent global health challenges, the importance of understanding airborne transmission within densely populated structures such as office towers has been highlighted [1]. This heightened awareness has precipitated a paradigm shift, emphasizing the paramount importance of indoor air quality. Emerging from this exigency is the innovative Mimosa kinetic façade, a design inspired by the adaptive nature of the *Mimosa pudica* plant.

The *Mimosa pudica*, which is renowned for its outstanding responsiveness to environmental stimuli, offers a natural model for the adaptation of the kinetic façade. Traditional architectural features frequently obstruct natural ventilation because of their static façade [2,3]. This results in problems such as reduced air quality, which can worsen respiratory conditions and decrease mental abilities. Due to its dynamic adaptability, the Mimosa kinetic façade has the potential to completely transform this area by providing effective airflow, eliminating airborne contaminants, and keeping a consistent indoor temperature. In addition to its practical advantages, this façade provides a distinctive architectural component that gives buildings a unique aesthetic identity. Cross ventilation is emphasized in this design and is a crucial strategy for improving airflow, especially in areas with high occupancy. This reduces dependency on mechanical systems by purifying the air and assisting in temperature control [4,5].

This study underscores the pivotal role of a building’s façade in enhancing the quality of life for its occupants. The primary emphasis is on how the effectiveness of the façade contributes to the wellness and wellbeing of those living within the space. As a subsequent outcome, an effective façade not only ensures the comfort and health of its inhabitants, but also bestows the building with architectural beauty. The project uses the biomimicry design spiral, a process drawn from nature, to imagine a façade that promotes cross ventilation [6,7]. By imitating natural processes, this methodical approach ensures innovative, efficient, and sustainable design outcomes.

This research addresses the critical need to mitigate airborne transmission in indoor environments. It underscores the importance of kinetic façades, particularly those inspired by the Mimosa plant. These façades not only improve indoor air quality but also contribute a unique aesthetic to buildings. Transitioning from innovative concepts to essential elements, these façades are increasingly recognized as key to ensuring safer indoor environments amid escalating airborne health challenges.

## 2. Literature Review

### 2.1. Related Papers

In this section, we synthesize the research gaps identified in various studies pertaining to both façade design and ventilation, with the objective of contextualizing the contributions of the Mimosa kinetic façade research. This synthesis is instrumental in bridging the identified gaps, thereby underpinning the significance of the proposed research.

**Façade Design Research:** In the realm of façade design and building architecture, numerous studies have pinpointed specific areas that necessitate further inquiry. The research on “Post-Occupancy Evaluation for Adaptive Façades [8]” underscores a notable absence of methodologies for assessing user interaction and satisfaction. Similarly, the study “Access to Daylight and View in Office Spaces [9]” acknowledges constraints arising from limited, potentially homogeneous participant groups, advocating for more inclusive and longitudinal investigations. A review on “Kinetic Façade Design for Visual and Thermal Comfort [7]” draws attention to the dearth of empirical data and the imperative for environmental impact evaluations. The paper titled “Performance Assessment of Adaptive Façade Systems [10]” identifies a lacuna in economic analysis and an incomplete understanding of the influence of user behavior on façade performance. Furthermore, the study “Adaptive Façade Systems [11,12,13]” emphasizes the paucity of information regarding market demands and the complexities entailed in integrating structural and energy aspects.

Moreover, research such as “Kinetic Facades in Hot, Dry Climates [14]” and “High Performance Building Façade Solutions [15]” exposes geographical focus limitations and the challenges of integrating these systems with existing building infrastructures, respectively. The paper “Adaptive Façade Definitions and Terms [16]” reveals an absence of comprehensive empirical data and a lack of focus on long-term sustainability. In “Solar Cooling Integrated Façades [17]”, researchers note a scarcity of real-world data and economic analysis. The study “Manufacturing Adaptive Façades with Standard Products [18]” points to a narrow focus on specific case studies and a dearth of real-world performance data.

Research studies on “Active Dynamic Windows for Buildings [19]” and “Automated Dynamic Facades—User Satisfaction and Interaction [20]” both identify gaps in real-world performance data and the need for extensive studies on long-term user perceptions. The paper on “Innovative Responsive Façade Elements Using Building Performance Simulation [21]” discusses a theoretical focus that lacks practical application data. Studies such as “Dynamic Façade Shading Typologies [22]” and “Transparent Building Envelopes—Innovative Technologies [23]” highlight limitations in shading position considerations and characterizations of dynamic performance, respectively.

Additionally, “Performance Prediction with Responsive Building Elements (RBEs) [24]” and “Energy Performance of Multiple-Skin Facades [10]” emphasize a focus on specific case studies and geographical limitations. The article on “Responsive Carrier Component Envelope (RCCE) [25]” and the paper “Geometry in Shading Systems and Biological Role Models [6]” accentuate structural challenges and theoretical discussions devoid of empirical validation. Papers such as “Thermal Performance of Active Envelopes [26]” and “Kinetic Photovoltaic Architecture in Performative Design [27]” discuss issues related to practical feasibility and long-term durability. “Climate Adaptive Building Shells (CABS) [28]” and a second study on “Automated Dynamic Facades—User Interaction [20]” reveal gaps in economic analysis and context-specific findings.

Finally, the study “Biology and Architecture Hybridization in French Architectural Offices [29]” indicates geographic limitations and a qualitative emphasis. Collectively, these gaps across various studies underscore the need for a multidisciplinary approach integrating user-centric design, technological innovation, environmental sustainability, and economic feasibility, which are vital for advancing the field of façade design and building architecture.

**Ventilation Research:** The selected studies on ventilation in the context of COVID-19 offer valuable insights but also present several limitations. For example, the study on infection risks in a Shenzhen outpatient building is confined to a specific healthcare context [30] and overlooks variables such as ventilation systems and occupant behavior. Research focusing on ventilation and air disinfection in office buildings [31] is predominantly simulation-based, lacking a thorough assessment of cost, safety, and environmental impact. The study on double-glazed façades with louvers in Isfahan, Iran [32] is geographically specific and reliant on static simulations. Investigations into cough droplet dynamics in hospital isolation rooms [33] and the role of ventilation in a German nursing home during a COVID-19 outbreak [34] are limited by their dependence on computational models and a lack of definitive evidence, respectively. The study of aerosol control in gyms during the pandemic [35] is hindered by a small sample size and does not conclusively link aerosol reduction to a decreased transmission risk. The review focusing on the physical office workplace and mental health [36] emphasizes the necessity for more comprehensive research methodologies. Agent-based simulations for epidemic risk assessment [37] and ventilation recommendations for COVID-19 mitigation [38] lack real-world validation and specificity. Finally, strategies for improving indoor air quality in HVAC systems during the COVID-19 pandemic [39] and the impact of airborne fine particle pollution on natural ventilation in Asian megacities [40] are constrained by their lack of practical case studies and geographical focus. These studies highlight the importance of broader, more practical, and diverse research for enhancing the effectiveness of ventilation strategies in various settings during pandemics.

**Mimosa Kinetic Façade: A Convergence of Research Gaps:** The “Mimosa Kinetic Façade: Bio-Inspired Ventilation Leveraging the Mimosa Pudica Mechanism for Enhanced Indoor Air Quality” addresses several identified gaps in both façade design and ventilation research. Regarding façade design, this biomimetic kinetic façade, inspired by the *Mimosa pudica* plant, not only considers aesthetic and structural factors, but also dynamically responds to environmental stimuli to optimize natural ventilation. This directly addresses the need for empirical data, environmental impact assessments, the integration of structural and energy aspects, and real-world performance data. The design’s responsiveness to environmental conditions positions the Mimosa kinetic façade as a user-centric and environmentally attuned solution.

In ventilation research, which is particularly relevant during the COVID-19 pandemic, the Mimosa kinetic façade introduces an innovative approach to natural ventilation, potentially mitigating airborne contaminants indoors. This addresses concerns about the lack of comprehensive assessments of ventilation systems and occupant behavior, the over-reliance on computational models, and the need for more practical and diverse research to enhance ventilation effectiveness. Utilizing the natural folding and unfolding mechanism of the *Mimosa pudica*, the façade design aims to improve air change rates and reduce CO${}_{2}$ levels, which are critical for addressing airborne transmission risks.

Furthermore, this research article details both computational simulations and physical tests to evaluate the façade’s impact on indoor air quality, responding to the call for more empirical data and the validation of theoretical models. This approach aligns with the demand for multidisciplinary research that not only considers user comfort and satisfaction, but also encompasses technological innovation and environmental sustainability.

In summary, this research article presents a holistic solution that fills identified research gaps by integrating responsive design elements, advancing empirical validation, and enhancing ventilation strategies, which are all crucial for the progression of façade design and the broader field of building architecture.

### 2.2. Ventilation Effect (Cross Ventilation)

**Figure 1:**
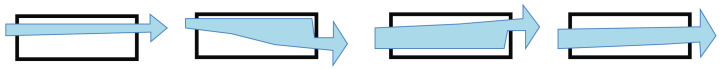
Impact of opening locations on cross-ventilation effect.

The concept of ventilation, particularly cross ventilation, has long been recognized as an effective method for enhancing indoor air quality. Cross ventilation allows air to flow seamlessly from one side of a structure to another, replacing indoor air with fresher outdoor air as shown in Figure 1. This natural process not only disperses airborne contaminants such as dust and allergens, but also prevents the buildup of stagnant air, ensuring a healthier environment [41].

Drawing inspiration from nature, the research introduces a kinetic façade modeled after the *Mimosa pudica*, a plant known for its responsive movements. Just as the *Mimosa pudica* reacts to external stimuli by folding its leaves, the kinetic façade is designed to adapt to changing environmental conditions, optimizing the flow of air into the working space [2,3].

By integrating the principles of cross ventilation into this innovative façade design, several benefits are realized. The continuous flow of air is maintained, ensuring that airborne particles are effectively dispersed. As a passive ventilation method, the façade is energy efficient, reducing the reliance on mechanical systems that might recirculate contaminants. Furthermore, the design aids in maintaining lower indoor CO${}_{2}$ levels, which is crucial for the wellbeing and productivity of occupants. The kinetic façade also addresses humidity concerns by allowing moist indoor air to be replaced with drier outdoor air, preventing mold growth and further enhancing air quality [41,42].

Beyond air quality, this kinetic façade, which is inspired by *Mimosa pudica* and utilizes cross ventilation, offers thermal benefits. It naturally cools the working space, reducing the need for energy-intensive air conditioning systems and providing a comfortable environment for occupants [43].

#### Enhancing Building Ventilation: The Synergy of EBC Annex 62 and the Mimosa Kinetic Façade

The integration of EBC Annex 62 principles into the cross-ventilation concept, particularly via the application of the Mimosa kinetic façade inspired by the *Mimosa pudica* plant, significantly enhances the efficacy of natural ventilation strategies in architectural design. Annex 62 underscores the critical role of ventilative cooling, a concept that is seamlessly actualized via the dynamic adaptability inherent in the Mimosa kinetic façade. This façade, mirroring the responsive movements of the *Mimosa pudica*, is adept at optimizing airflow in response to varying environmental conditions, a strategy that resonates with the core tenets of Annex 62. It dynamically modulates in order to enable cross ventilation, facilitating the unimpeded flow of fresh air throughout the building, thereby supplanting the indoor air with a cleaner, outdoor counterpart. This mechanism is in direct alignment with the objectives of Annex 62, which advocates for the utilization of natural ventilation as a means to enhance indoor air quality while concurrently reducing energy consumption [44].

Incorporating the VC tool, as delineated in Annex 62, allows for precision tuning of the façade’s functionality, leveraging real-time climatic data and occupancy patterns. This tool is instrumental in evaluating the effectiveness of ventilative cooling, directing the façade’s adjustments to optimize cross ventilation under favorable conditions. Such strategic operation ensures the maintenance of a comfortable indoor environment, characterized by diminished levels of airborne particles and CO${}_{2}$, and effectively addresses issues of humidity and thermal comfort.

Moreover, the amalgamation of Annex 62 principles with the Mimosa kinetic façade fosters the development of energy-efficient buildings. This synergy significantly reduces the dependence on mechanical ventilation and air conditioning systems, thereby contributing to a reduction in energy consumption and operational expenses. This approach not only augments indoor air quality and occupant comfort, but also aligns with the sustainable building practices and energy efficiency standards emphasized in Annex 62.

In essence, the application of EBC Annex 62’s ventilative cooling strategies via the Mimosa kinetic façade presents a sophisticated, biomimetic solution to the challenges of indoor air quality and thermal comfort in modern buildings. This integration culminates in edifices that are not only conducive to the health and comfort of occupants, but are also emblematic of energy efficiency and environmental stewardship.

### 2.3. Human Comfort

“Human comfort” is a multifaceted term that encapsulates the physical and psychological wellbeing of individuals in relation to their environmental conditions. It includes thermal comfort, influenced by temperature, humidity, and air movement; visual comfort, concerning the adequacy of lighting and glare reduction; acoustic comfort, focusing on ambient noise levels and their impact on activities; ergonomic comfort, relating to the physical interaction with the environment; and psychological comfort, which involves feelings of safety, privacy, and overall environmental satisfaction. This concept is integral to the fields of ergonomics, environmental psychology, and architectural design, with the objective of enhancing health, productivity, and wellbeing in both living and working spaces [45].

Central to this study is the aspect of thermal comfort, particularly the role of wind speed in indoor environments. Research indicates that a wind speed range of 1 to 5 m/s is considered comfortable, balancing air movement to avoid feelings of stagnation while preventing discomfort from drafts or excessive cooling. Below 1 m/s, air can feel stuffy and lead to increased perceived temperatures and discomfort, while speeds above 5 m/s can be uncomfortable or even dangerous, causing drafts, cooling, and disturbances. Therefore, maintaining indoor air movement akin to an outdoor wind speed of 1 to 5 m/s is crucial for optimal human comfort [46] as shown in Table 1. Maintaining a balance between indoor environmental quality and energy efficiency is crucial in the design of heating, ventilation, and air conditioning (HVAC) systems, as well as in planning natural ventilation in buildings. This balance is essential for creating environments that promote health and wellbeing. In the context of Bangkok, Thailand, this consideration becomes even more significant due to the city’s tropical monsoon climate. Bangkok is characterized by year-round warm temperatures, typically ranging from 25 ${}^{\circ }$C to 35 ${}^{\circ }$C, and high humidity levels, often exceeding 70%. The city undergoes a rainy season from May to October, influenced by the southwest monsoon, which brings heavy rainfall and stronger winds. Conversely, the dry season, from November to April, is marked by lighter winds. These climatic factors, including moderate wind speeds averaging 2 to 5 m per second, play a significant role in influencing the design and effectiveness of kinetic façades in the region. Such façades are integral to achieving the optimal balance in indoor climate control, ensuring that buildings are both comfortable and energy efficient.

### 2.4. Indoor Air Quality (IAQ)

Indoor air quality (IAQ) is crucial in enclosed spaces such as homes and offices, particularly during health crises when traditional ventilation methods may be inadequate [38,39]. This study explores the development of advanced façades that modulate ventilation rates based on room size and occupancy, with a focus on maintaining CO${}_{2}$ levels below 700 ppm to optimize air quality [47] as shown in Table 2. These façades, which blend contemporary design with sustainable architecture, either enhance natural ventilation, integrate with HVAC systems, or dynamically adjust as kinetic façades using real-time data.

A critical measure for evaluating these façades is the air change rate (ACR), quantified in air changes per hour (ACH). The ACR is essential for assessing the efficacy of ventilation in diluting airborne particles, including viruses such as COVID-19. For example, an ACH of 1 indicates complete air replacement within an hour. The American Society of Heating, Refrigerating, and Air-Conditioning Engineers (ASHRAE) recommends a minimum outdoor air ventilation rate of 5 cubic feet per minute per person, or 0.06 per square foot for offices [48,49], with residential buildings typically requiring an ACR of 0.35. However, office spaces may necessitate an ACH of 2–3, particularly for mitigating airborne diseases [50].

The study involves measuring the ACR of kinetic façades using methods such as direct airflow rate measurements and computational fluid dynamics simulations. Comparing the achieved ACR against ASHRAE standards is crucial. If kinetic façades yield higher ACR values, enhanced ventilation is indicated, potentially reducing airborne contaminants and significantly improving IAQ. This research underscores the importance of integrating an innovative façade design in building architecture to promote healthier indoor environments.

## 3. Methodology

### 3.1. Research Methodology Overview

**Figure 2:**
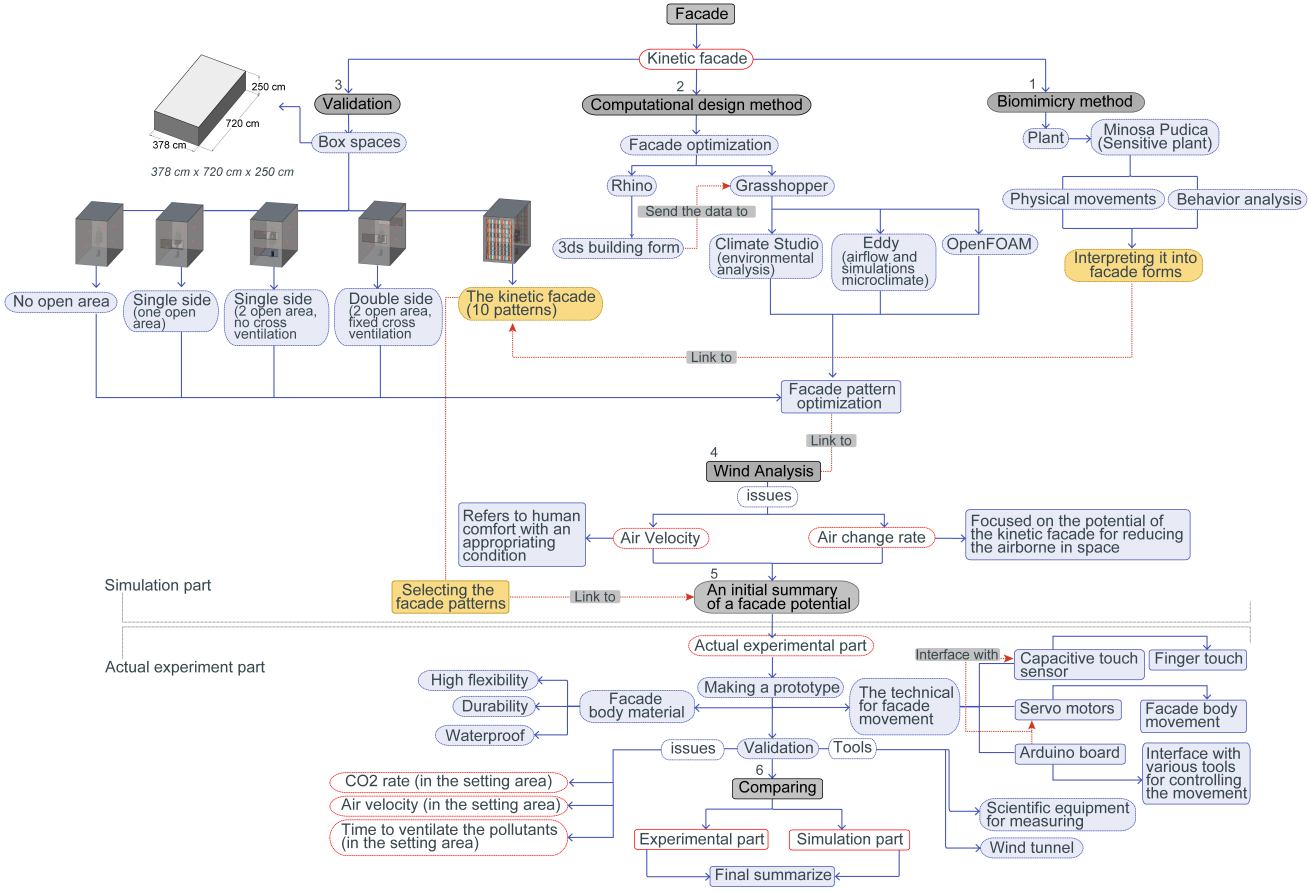
Flowchart illustrating the research methodology employed in this study.

Figure 2 illustrates the research methodology used for this study on kinetic façades inspired by the *Mimosa pudica* plant. It is structured into six distinct parts:

1. Biomimicry Method: This involves creating the kinetic façade by emulating the physical movement and behavior of the *Mimosa pudica*. The façade’s design is derived using biomimicry principles.
2. Computational Design Method: This focuses on façade optimization using Rhino 7 for 3D modeling and Grasshopper for environmental analysis (Climate Studio), as well as air velocity and CO${}_{2}$ concentration analysis (Eddy 3D and OpenFOAM (version2212)). This step also includes optimizing façade patterns.
3. Validation: This uses a simulated box space (378 cm × 480 cm × 250 cm) to validate different façade types, including the Mimosa-pudica-inspired kinetic façade, assessing their impact on ventilation and indoor CO${}_{2}$ levels.
  The room dimensions of 378 cm × 480 cm × 250 cm, totaling 27.216 sq.m, were chosen for the simulation for several reasons. Firstly, these dimensions closely resemble the common room sizes found in real-world settings, such as offices, enabling the simulation to realistically depict practical scenarios. Secondly, the ample size of the room is crucial for evaluating the effectiveness of various façade designs in enhancing ventilation and reducing indoor CO${}_{2}$ levels. Thirdly, these specific dimensions are optimized for computational modeling, achieving a balance between realistic space representation and computational resource efficiency. Fourthly, the room is spacious enough to clearly demonstrate the effectiveness of different façade designs in improving ventilation, especially the Mimosa-pudica-inspired kinetic façade. Lastly, the insights obtained from simulations conducted in this room will provide a solid baseline for future comparative analyses with different room dimensions, contributing to a comprehensive understanding of the impact of façade designs on ventilation. This meticulous selection of room dimensions ensures that the simulation results will be both highly relevant and applicable, thereby enhancing the overall validity and utility of the study.
4. Wind Analysis: This examines how air velocity affects human comfort and the air change rate (ACR) to evaluate the kinetic façade’s effectiveness in reducing airborne contaminants.
5. Initial Summary: Post-simulation, a prototype is developed, incorporating materials and mechanisms such as capacitive touch sensors, servo motors, and an Arduino board. The prototype undergoes validation in a wind tunnel, focusing on the CO${}_{2}$ rate, air velocity, airborne particle concentration, and ventilation time.
6. Comparison: This compares simulation results with real-world test outcomes to conclude the kinetic façade’s efficacy in enhancing air ventilation and indoor air quality.

This methodology combines computational and experimental approaches to assess the potential of a biomimicry-inspired kinetic façade in improving indoor environmental conditions.

### 3.2. Variable

This research on the kinetic façade, inspired by the *Mimosa pudica* concept, comprises computational simulations and actual testing. The simulations explore a wide range of variables, while the actual tests prioritize façade selections from simulations in order to compare them with static façade cases. Both methods aim to assess the façade’s efficacy in enhancing ventilation and indoor air quality. The variables for the two sections are shown in Table 3:

### 3.3. Simulation Part: The Façade Type for Simulation

**Figure 3:**
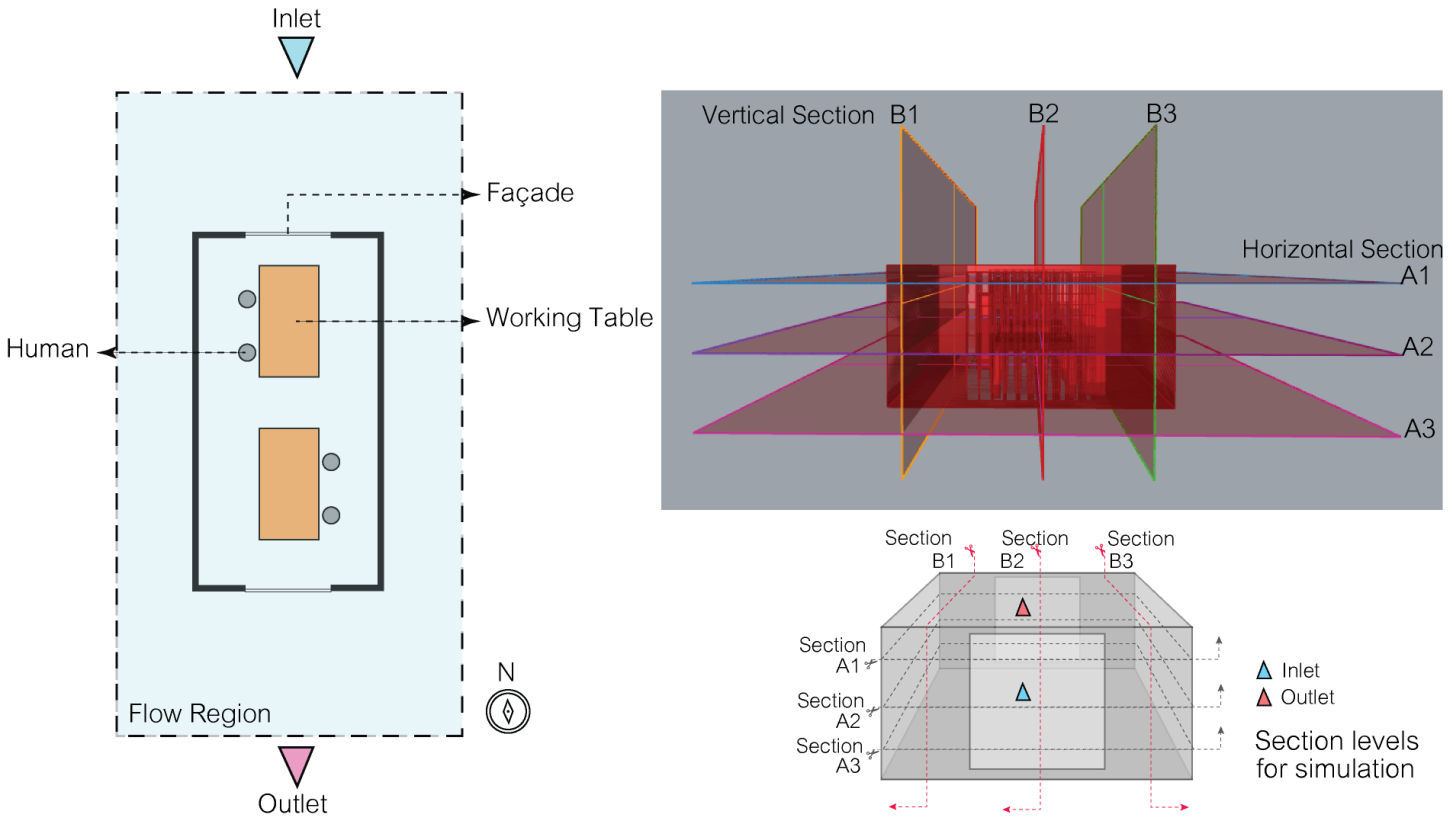
Determined plan and section for simulation.

The simulation of the kinetic façade inspired by the *Mimosa pudica* is an essential step before moving to prototype testing, serving multiple key functions. It provides a preliminary evaluation of the façade’s effectiveness in reducing airborne contaminants, allowing for optimization and risk mitigation. Additionally, it saves time and costs by allowing for digital refinements, leading to better-informed decisions regarding the design and performance. The research considers various aspects of airflow within the workspace, dividing the section plane into six positions, as shown in Figure 3, and focusing on elements such as airflow velocity and air change rate (ACR) for a comprehensive understanding of the façade’s potential performance.

**Figure 4:**
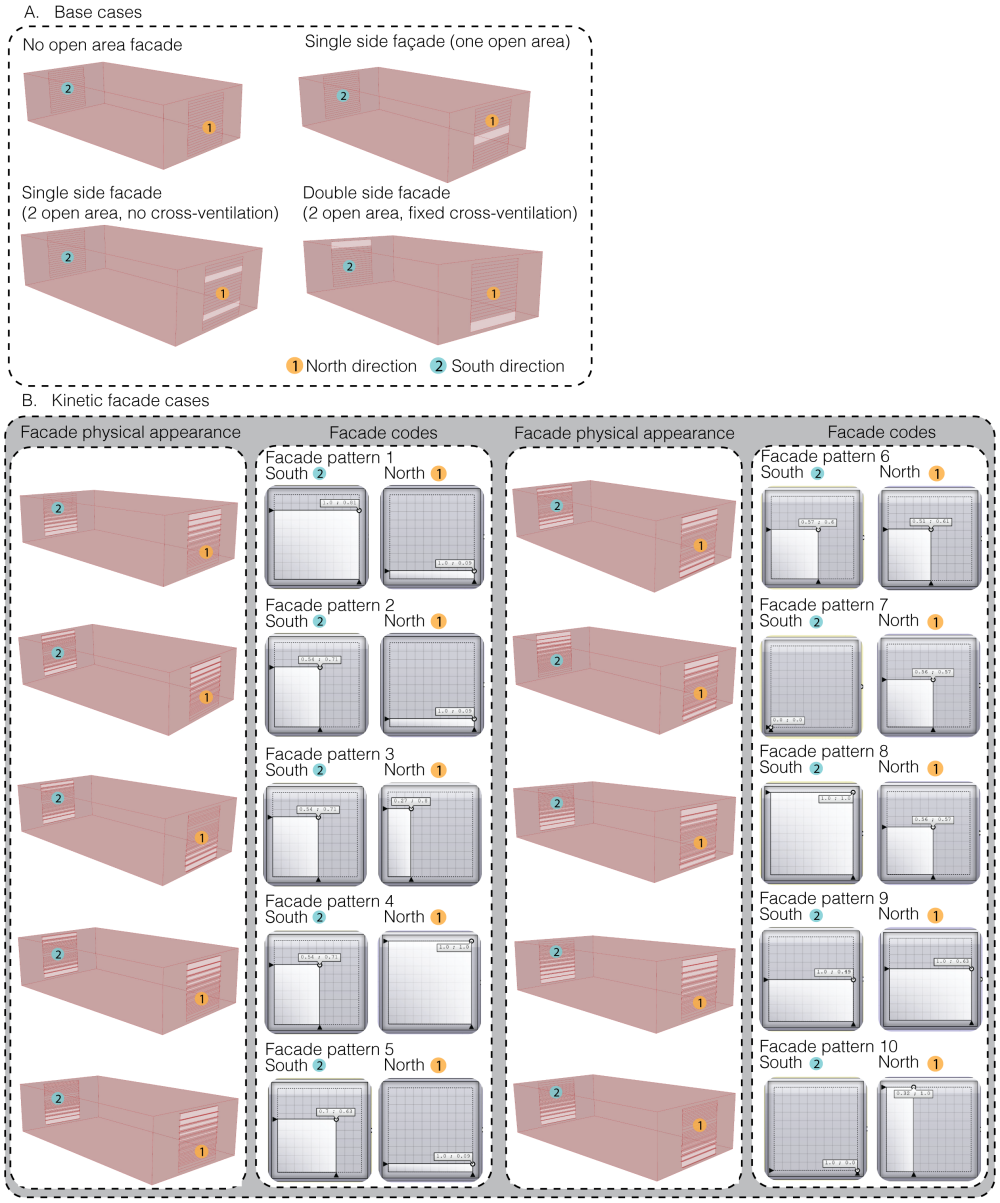
Comparative visuals of static façades ((**A**) Base cases) and bio-inspired façades ((**B**) Kinetic façade cases): this illustration contrasts established façade types with ten distinct kinetic patterns, each inspired by the adaptive *Mimosa pudica* plant.

In this study, two façade designs were compared: a standard version, as depicted in Figure 4 (A. Base cases), and another design inspired by the Mimosa plant that showcases ten different patterns, as also shown in Figure 4 (B. Kinetic façade cases). The relationship between the façade’s physical appearance and its code is defined by the kinetic façade codes. These codes, used in the Grasshopper software, dictate how the kinetic façade module opens, influencing the cross-ventilation effect. Different codes result in varied façade patterns, as reflected in their physical appearance. The research aimed to assess the kinetic façade’s influence on air circulation. Emphasizing cross ventilation, where cool air enters one side and warm air exits the opposite, is essential for air quality. Façade patterns on the north and south should be staggered, not grid-aligned, to enhance airflow. The design must also account for factors such as wind direction, sun orientation, and weather to ensure optimal ventilation and a comfortable workspace.

### 3.4. Actual Experiment Section: The Static vs. Kinetic Façades

**Figure 5:**
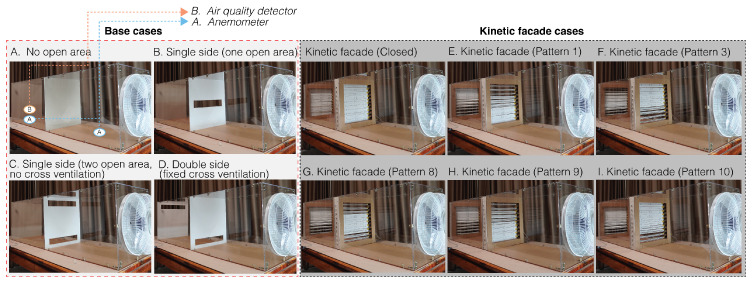
Experimental patterns of the static and kinetic façades.

- The static façades (A–D)—base cases: These represent traditional or conventional façade designs as shown in Figure 5. Testing these designs provides a baseline against which the performance of kinetic façades can be compared. It is essential to understand how much improvement or difference kinetic façades offer over traditional designs.
- The kinetic façades (E–I)—bio-inspired façades: These are dynamic façades whose designs were selected based on simulations as shown in Figure 5. The idea is to evaluate if the simulated potential of these façades translates effectively into real-world scenarios.

## 4. Biomimicry Method

### 4.1. Applying the Biomimicry Approach to the Concept of a Kinetic Façade

**Figure 6:**
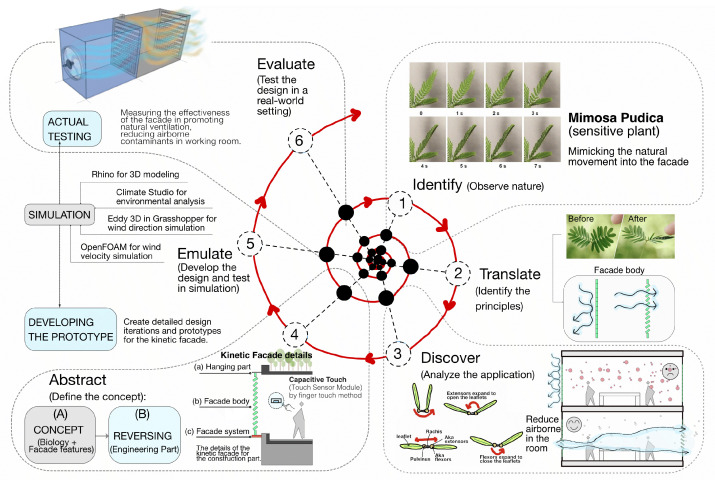
The biomimicry approach to the concept of a kinetic façade.

Biomimicry draws inspiration from nature to develop sustainable solutions for challenges that humans face [51]. It seeks to harmonize our environment with the natural world, fostering a symbiotic relationship between humans and nature [52]. Carl Hastrich pioneered the biomimicry design spiral in 2005 [51,53], outlining a systematic six-step approach to transform nature’s innovations into sustainable human designs. As Figure 6 shows, this methodology is exemplified by the kinetic façade concept, inspired by the *Mimosa pudica* plant. This innovative design aims to enhance indoor workspaces by applying the principles of biomimicry, translating nature’s systems into practical human applications via a structured six-step process [53,54].

1. Identify (observe nature): Study the *Mimosa pudica* plant, the unique folding and unfolding behavior of its leaves, and the mechanisms behind its responsive movement to external stimuli, focusing on their relevance to types of office buildings.
2. Translate (identify the principles): Understand how the plant’s behavior can be translated into a kinetic façade system with three distinct elements (hanging part, façade body, and façade system) that reacts to environmental conditions (e.g., air quality, temperature, and humidity) to optimize natural ventilation and improve indoor air quality in working spaces.
3. Discover (analyze the application): Investigate the potential applications of the Mimosa-pudica-inspired kinetic façade in office buildings, with a focus on enhancing ventilation and reducing airborne contaminants such as CO${}_{2\;}$ and COVID-19 in working rooms.
4. Abstract (define the concept): Develop a conceptual design for the kinetic façade, integrating the principles of the *Mimosa pudica* plant, the identified applications, and the three façade elements (hanging part, façade body, and façade system) in a functional, aesthetically pleasing, and structurally sound manner.
5. Emulate (develop the design and test in simulation): Create detailed design iterations and prototypes of the kinetic façade using computational methods, such as Rhino for 3D modeling, Climate Studio for environmental analysis, Eddy 3D in Grasshopper for wind direction simulation, and OpenFOAM for wind velocity simulation, focusing on the performance in office building environments.
6. Evaluate (test the design in a real-world setting): After the simulation phase, construct a physical prototype or scaled model of the kinetic façade under controlled environmental conditions in a working space (office building type) to assess its performance in a real-world scenario. This step should include measuring the effectiveness of the façade in promoting natural ventilation, reducing airborne contaminants, and improving overall indoor air quality in working rooms.

By following the six-step biomimicry method, the behavior of the *Mimosa pudica* can be translated into a kinetic façade system that promotes healthier and more sustainable indoor environments in workspaces.

### 4.2. The Process of Mimicking the Mimosa Pudica Movement

**Figure 7:**
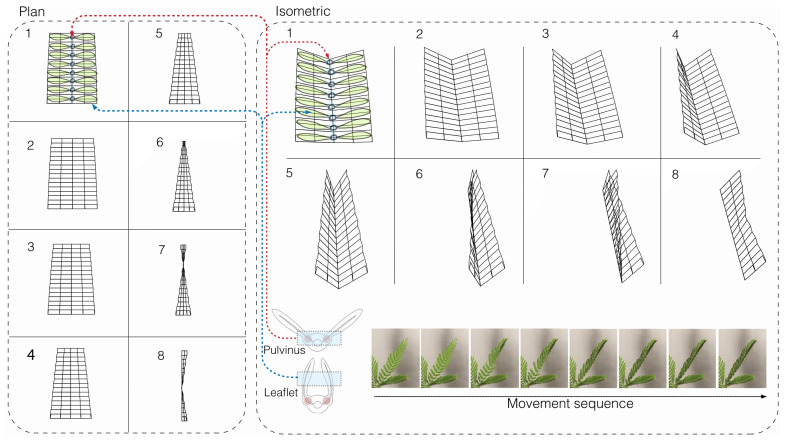
Sequential illustration of the *Mimosa pudica*’s movement in eight steps. A parametric model was created in Grasshopper, using this digital representation to match the movements of the plant as shown in Figure 7. The numbers from 1 to 8 represent the leaflet movement at each step.

**Figure 8:**
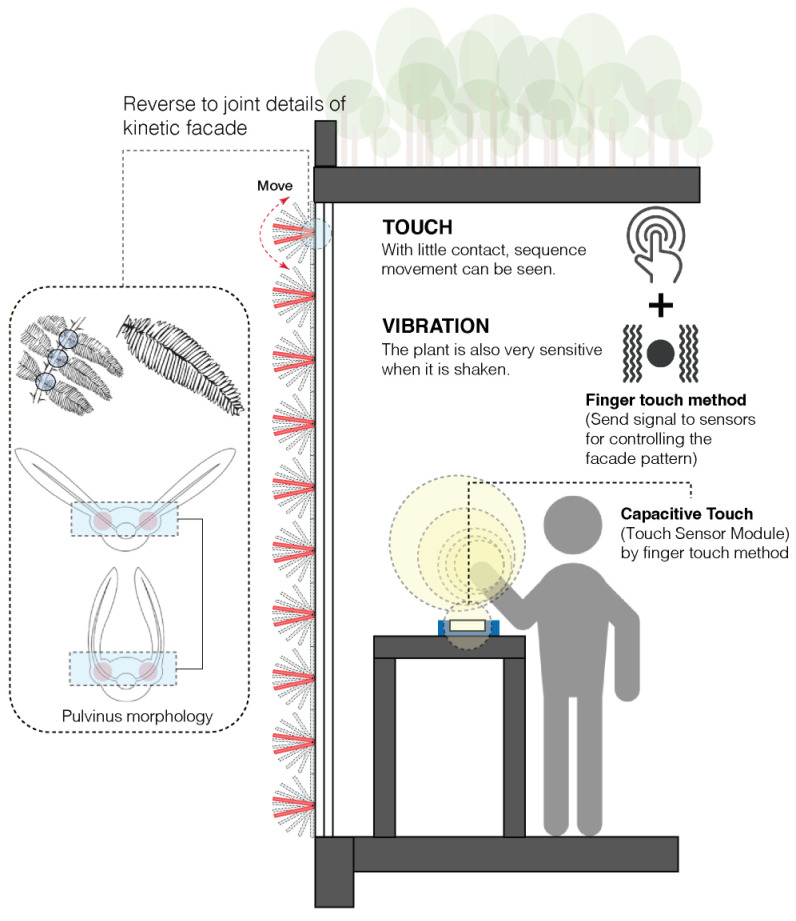
Details of the kinetic façade interpreted from the *Mimosa pudica*.

Translating the movement of the *Mimosa pudica* into a digital form involves a series of multiple stages. Initially, the study began by meticulously observing the movements of the *Mimosa pudica* plant as it responded to stimuli. For the sake of clarity, this observation was divided into several sections. A 3D digital model of the *Mimosa pudica* was created using Rhino, emphasizing its moving parts. A parametric model was created in Grasshopper using this digital representation to match the movements of the plant as shown in Figure 7. In order to control the folding and unfolding of the digital model and mimic the behavior of the real plant, a sequence was built in Grasshopper. Sensing devices such as capacitive touch were added into the model to increase realism, and actuators were integrated to control movement as shown in Figure 8. This allowed the façade to mimic the behaviors of the *Mimosa pudica*. The digital model was rigorously tested and improved in the last step to maximize its responsiveness and effectiveness.

## 5. Experiment and Results

### 5.1. Simulation Part: The Results of Air Velocity in the Space

During the simulation phase, the façade’s effectiveness was assessed using two primary metrics. Firstly, air velocity, which pertains to the speed of indoor air circulation, plays a pivotal role in both human comfort and the reduction in airborne contaminants. Secondly, the air change rate (ACR) measures the frequency with which outdoor air replaces indoor air in a workspace, which is a vital metric for ensuring optimal indoor air quality and minimizing health risks. Through these simulations, the goal is to identify the most promising façade patterns for subsequent validation in actual experimental procedures.

The accurate measurement of air velocity is crucial for ensuring human comfort in both indoor and outdoor environments. Adequate air movement is essential for maintaining good indoor air quality and thermal comfort, while excessive wind speeds can lead to discomfort. Proper design and assessment of ventilation systems play a crucial role in maintaining optimal air velocity levels for human comfort as shown in Table 1. In the present study, the focus was on evaluating the efficacy of different façade types in this regard, and simulation was employed as the primary method used to assess their performance.

In this study, the air velocity ranges associated with various façade designs were evaluated to determine their efficacy in ventilating airborne particles in indoor spaces and maintaining human comfort. The assessment took into account the climatic conditions in Bangkok, Thailand.

**Figure 9:**
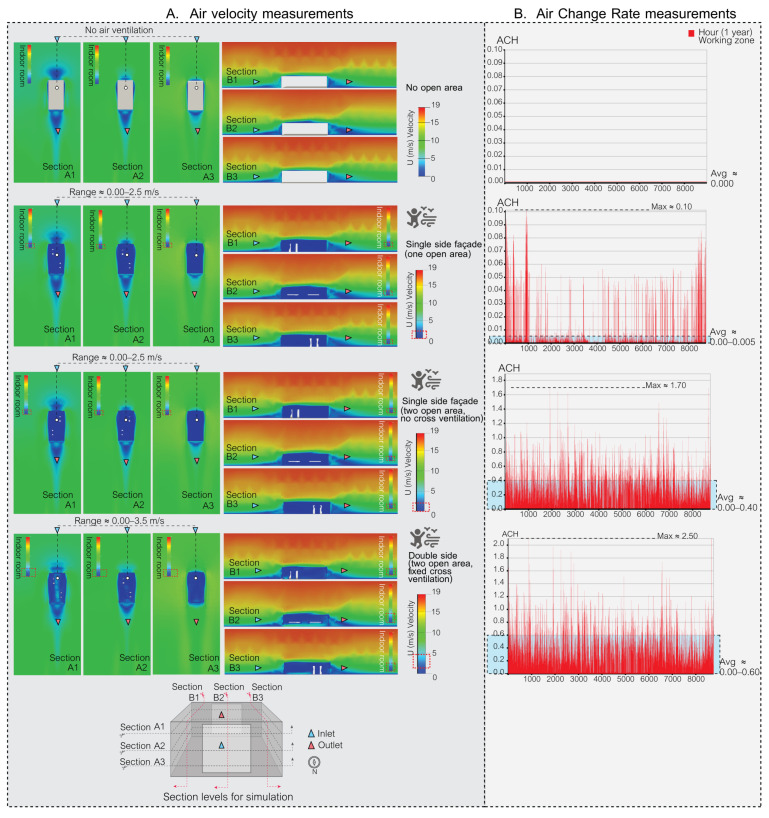
Air velocity and air change rate measurements in a space with base cases (no open areas), single-sided façade (one open area), single-sided façade (two open areas, no cross ventilation), and double-sided façade (two open areas, fixed cross ventilation).

**Figure 10:**
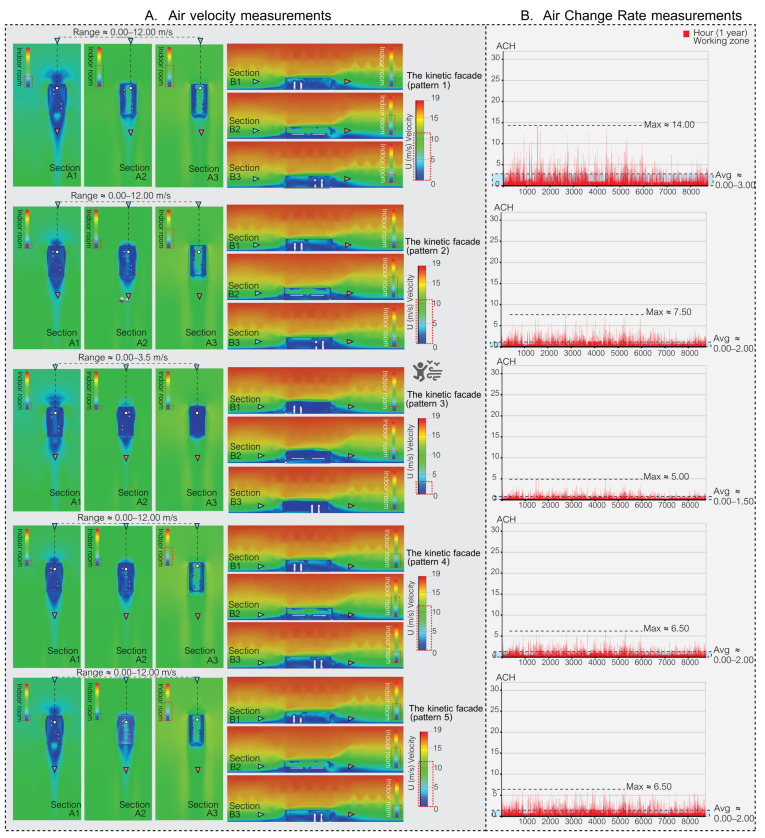
Air velocity and air change rate measurements in a space featuring Mimosa-inspired façade patterns 1, 2, 3, 4, and 5.

**Figure 11:**
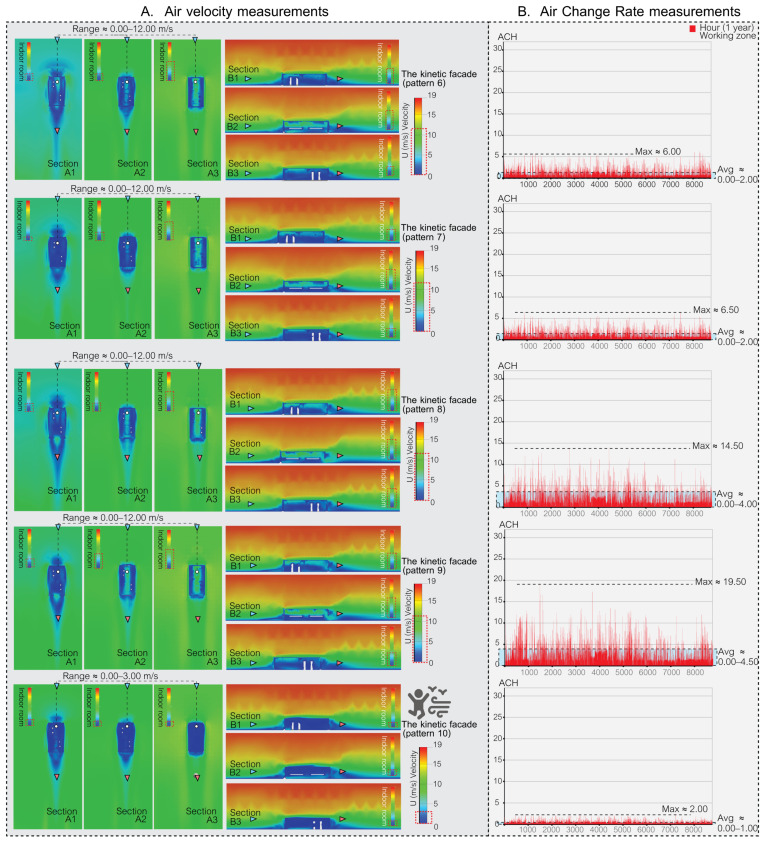
Air velocity and air change rate measurements in a space featuring Mimosa-inspired façade patterns 6, 7, 8, 9, and 10.

The results are summarized as follows:

1. Figure 9 depicts a space with no open areas, resulting in a complete absence of air ventilation and making the space unsuitable for human habitation. In contrast, Figure 9 also presents spaces with single-sided openings and two open areas (with and without cross ventilation), where air velocities ranged from 0.00 to 2.50 m/s. While these configurations ensured comfort for occupants, they were not effective in ventilating airborne particles.
2. Figure 10 and Figure 11 illustrate kinetic façades inspired by *Mimosa pudica* (patterns 1, 2, 4, 5, 6, 7, 8, and 9); they demonstrated the ability to ventilate airborne particles with air velocity ranges between 0.00 and 12.00 m/s. Nevertheless, these designs may cause discomfort for occupants over prolonged periods.
3. Figure 10 and Figure 11 illustrate Mimosa-inspired kinetic façades with patterns 3 and 10, which provided occupant comfort (air velocity ranges of 0.00–3.50 m/s and 0.00–3.00 m/s, respectively). However, these designs showed limited efficacy in ventilating airborne particles.

As depicted in Table 4, air velocity measurements play a significant role in assessing human comfort. It can be deduced that achieving an optimal façade design necessitates the integration of elements from both effective and less effective ventilation patterns, thereby striking a balance between human comfort and airborne particle ventilation. Consequently, in order to select an appropriate pattern for the façade prototype, the research will focus on five specific patterns that exhibit distinct characteristics. Patterns 1, 8, and 9 of the kinetic façade are notably proficient in ventilating airborne particles within the space. On the other hand, patterns 3 and 10 of the kinetic façade are deemed suitable for facilitating prolonged human habitation within the enclosed environment. By meticulously considering the strengths and weaknesses of these patterns, a well-informed decision can be made regarding the most suitable choice for the façade prototype.

### 5.2. The Result of Air Change Rate in the Space

The air change rate per hour (ACH) serves as a vital metric for evaluating the potential of various façade designs in terms of air ventilation, which is crucial for mitigating airborne contaminants in enclosed spaces. This study conducted simulations over a full year, accounting for 8760 hourly data points, in a small office setting with a meeting room function in Bangkok, Thailand.

The American Society of Heating, Refrigerating, and Air-Conditioning Engineers (ASHRAE) recommends a minimum ACH of 2.00–3.00 for office spaces, while COVID-19-related recommendations for hospital areas vary depending on the function. In this research, a minimum ACH of 2.00 was set as a criterion for reducing airborne contaminants in the space using passive ventilation methods.

The ACH values for each distinct façade design are as follows.

Figure 9 outlines four distinct base case ventilation scenarios for comparison with the kinetic façade, each marked by different ACH values. The “no open area case” indicates that there is no air ventilation in the indoor space. In the “single side (one open area)” scenario, ventilation slightly improves, evidenced by a maximum ACH of 0.10 and an average ranging from 0.000 to 0.005. The scenario featuring a single side with two open areas but without cross ventilation exhibits a notable increase in ventilation, with a peak ACH of 1.70 and an average spanning 0.00 to 0.40. The most effective ventilation is observed in the double-sided case with two open areas and fixed cross ventilation, where the ACH peaks at 2.50 and the average varies between 0.00 and 0.60.

Figure 10 and Figure 11 showcase the enhanced ventilation performance of kinetic façade designs inspired by the Mimosa plant, featuring patterns 1 to 10. Patterns 1 to 7, as depicted in Figure 10, achieve maximum air change rate per hour (ACH) values ranging from 5.00 to 14.00, with average ACH values between 0.00 and 3.00. Figure 11 highlights pattern 8’s outstanding ventilation, with a peak ACH of 14.50 and an average ranging from 0.00 to 4.00. It also presents pattern 9’s exceptional ventilation capabilities, reaching a maximum ACH of 19.50 and an average between 0.00 and 4.50, while pattern 10 exhibits satisfactory performance with a maximum ACH of 2.00 and an average from 0.00 to 1.00. In conclusion, kinetic façades inspired by the Mimosa plant (patterns 1–10) show a higher potential for air ventilation and effectively reduce airborne particles in office spaces using passive ventilation methods. In particular, patterns 8 and 9 exceed the ASHRAE’s minimum criteria for office spaces. The study highlights the advantages of these innovative façade designs in enhancing air change rates and promoting healthier indoor environments for workspaces.

In Bangkok’s hot and humid climate, managing indoor air quality and comfort is challenging, particularly when the air change rate (ACR) exceeds 15, due to increased demands for cooling and dehumidification. However, innovative kinetic façade designs inspired by the Mimosa plant, especially pattern 9, show promise in effectively managing these challenges. While occasionally reaching a maximum ACH of 19.50, pattern 9 generally maintains an average ACH well within the comfortable range of 0.00 to 4.50. This performance suggests that, despite brief periods of a higher ACR, these façades can align with Bangkok’s climatic needs for a comfortable and energy-efficient indoor environment. Further enhancing this potential, a holistic façade prototype emerges by amalgamating the strengths of various Mimosa-inspired patterns. This prototype combines the comfort-centric attributes of patterns 3 and 10 with the superior airborne particle ventilation capabilities of patterns 1, 8, and 9. Such a unified façade design aims to create spaces that are not just habitable but offer a comprehensive living experience, balancing enhanced air velocities for occupant comfort with an optimal ACH for maintaining air quality. This approach contributes to a future where architectural innovation harmoniously blends human comfort with effective air quality management.

### 5.3. Actual Experiment Section: Evaluation of Façade Types Based on Ventilation Potential: A Study Using the Indoor Air Quality Index (IAQI)

This study emphasizes the value of real-world application and places the priority on actual evaluations over explicit simulations. The rate of airborne particle ventilation, air velocity for comfort, and CO${}_{2}$ content as an indicator of air quality are important testing criteria. Prototypes are tested in controlled environments to acquire a real understanding of how well the façade performs. This practical learning approach helps design modifications and strikes a balance between innovative ideas and practical implementation.

In the research, smoke fog oil was provided as a substitute for airborne particles. Before testing the ventilation capacities, this oil saturated the experimental area to a concentration of 6000 ppm. This decision was crucial because, despite being harmless, smoke fog oil mimics the dispersion and settlement tendencies of airborne viruses. Recent global health crises have heightened worries regarding airborne transmission, making this scenario all the more vital. By evaluating the behavior of smoke fog oil under the effect of the kinetic façade, it is possible to gain insight into the potential behavior of authentic airborne particles in similar environments.

**Figure 12:**
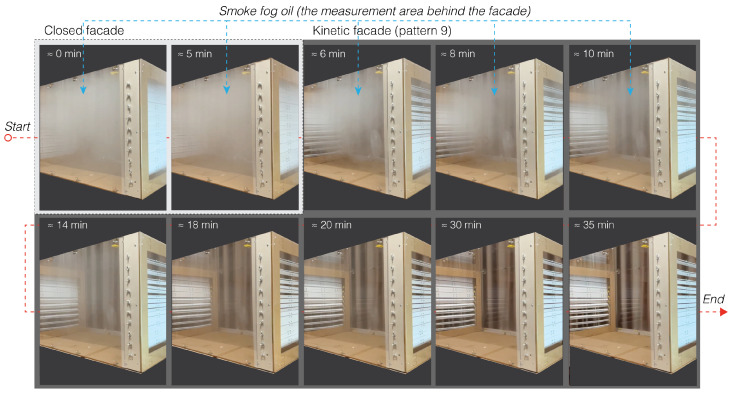
Observational analysis of airborne transmission within 30 min following façade opening utilizing kinetic façade (pattern 9).

The study broadened its examination to assess the impact of diverse façade designs on indoor CO${}_{2\;}$ reduction. CO${}_{2\;}$ levels, monitored in a controlled environment for 30 min, are depicted in Figure 12, with outcomes categorized as per the indoor air quality index in Table 2. An observational analysis highlighted the airborne transmission within 30 min after the opening of the façade using a kinetic façade (pattern 9). Figure 12 distinctly shows that, from 0 to 5 min (prior to façade opening), smoke density remains high indoors. Following the opening of the façade when employing kinetic façade pattern 9, there is a significant smoke reduction after 5 min, with the smoke nearly vanishing from 20 to 25 min.

The selected timeframe was determined based on multiple considerations: it reflects the usual duration that an individual might spend in a confined area; it permits several air exchange cycles typical of most ventilation systems; it is consistent with the duration adopted in comparable studies; and it provides a sufficient window for detecting notable CO${}_{2}$ variations while still being concise enough to retain experimental oversight. Essentially, this timeframe ensures that the study’s findings are both scientifically accurate and applicable to real-world situations.

**Figure 13:**
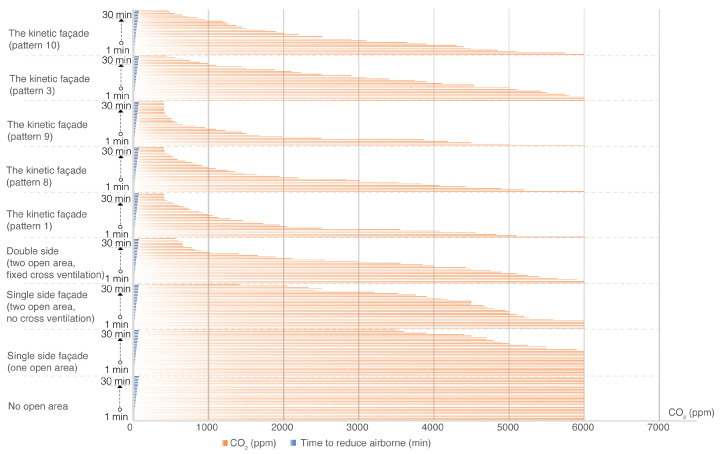
Comparative analysis of CO${}_{2}$ levels over time for different façade types.

Table 5 presents the rankings of façade types based on their CO${}_{2}$ concentration levels at the end of a 30 min evaluation period. Additionally, a comparative analysis of CO${}_{2}$ levels over time for different façade types is illustrated in Figure 13.

The kinetic façades, regardless of their specific patterns, emerged as the top performers in terms of ventilating airborne particles within the stipulated 30 min, all achieving a commendable “Good” IAQI rating. In contrast, the façade with no open area was markedly less effective, falling into the “Extreme” IAQI category. The study underscores the significance of open areas and cross ventilation in façade design for optimal indoor air quality.

#### Analysis of Air Velocity Concentrations across Various Façade Types over Time

**Figure 14:**
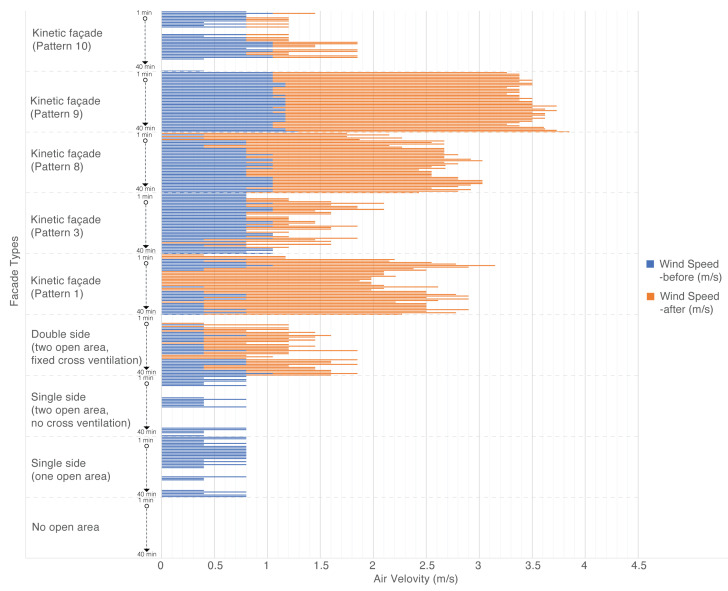
Comparative analysis of air velocity across different façade types.

The graph in Figure 14 illustrates the observed air velocities (m/s) over a span of 40 min for each façade type, subjected to standardized testing conditions.

The research employed a 40 min testing duration for air velocity to ensure consistent measurements across façade designs. This timeframe captures the façades’ steady behavior, reduces the impact of short-term disturbances such as gusts, and reflects real-world conditions. By reaching a quasi-steady state, especially under thermal influences, the study offers a balanced, accurate, and practical insight into the façades’ performance in indoor environments.

The study revealed that façades without open areas showed no air movement, registering a consistent velocity of 0 m/s. Similarly, single-sided façades, regardless of the number of open areas, failed to facilitate any significant air movement. Double-sided façades with fixed cross ventilation showed modest improvements, with velocities reaching up to 1.05 m/s. However, kinetic façades showcased the most promise. Pattern 1 peaked at 2.21 m/s, while pattern 3 achieved velocities of up to 1.05 m/s. Pattern 8 closely followed pattern 1, reaching 1.98 m/s. Impressively, pattern 9 emerged as the top performer, with velocities ranging between 2.21 m/s and 2.56 m/s. In contrast, pattern 10 lagged behind other kinetic designs, peaking at just 0.8 m/s. Overall, the research accentuates the pivotal role of architectural design in influencing ventilation efficiency, with kinetic façades, especially pattern 9, demonstrating superior potential in optimizing air movement within interior spaces.

In a comparative analysis of various façade types, it becomes evident that the design and structure of a façade significantly influence indoor air quality and ventilation efficiency. Static façades, such as those with no open areas or limited open areas, demonstrate restricted ventilation capabilities. For instance, façades with no open areas exhibit a complete absence of ventilation, leading to an “Extreme” IAQI rating due to elevated CO${}_{2}$ concentrations. Similarly, façades with a single open area, despite occasional peaks in wind speed, ultimately succumb to poor air quality, as indicated by their “Hazardous” IAQI rating. Conversely, kinetic façades, irrespective of their specific patterns, consistently outperform their static counterparts. These kinetic façades not only achieve “Good” IAQI ratings but also maintain efficient ventilation, even at varying wind speeds. Notably, certain kinetic façade patterns, such as pattern 9, demonstrate exceptional ventilation efficiency, with wind speeds reaching up to 2.56 m/s, while maintaining consistent CO${}_{2}$ concentrations comparable to other kinetic façades. In conclusion, the kinetic façades undeniably present a more promising potential in terms of ventilation and air quality when juxtaposed with static façades. Their dynamic design, coupled with multiple open areas, ensures optimal indoor air quality, underscoring their superiority in architectural design for sustainable and healthy living environments.

## 6. Discussion

The results indicate that kinetic façades, particularly patterns 1, 8, and 9, markedly surpass static façades (base cases) in ventilation efficiency. This significant finding underscores the practical applicability of these innovative designs, extending beyond theoretical expectations. Furthermore, patterns 3 and 10, though less effective in air purification, are identified as more suitable for prolonged human occupancy, emphasizing the necessity of balancing ventilation effectiveness with human comfort in façade design.

**Figure 15:**
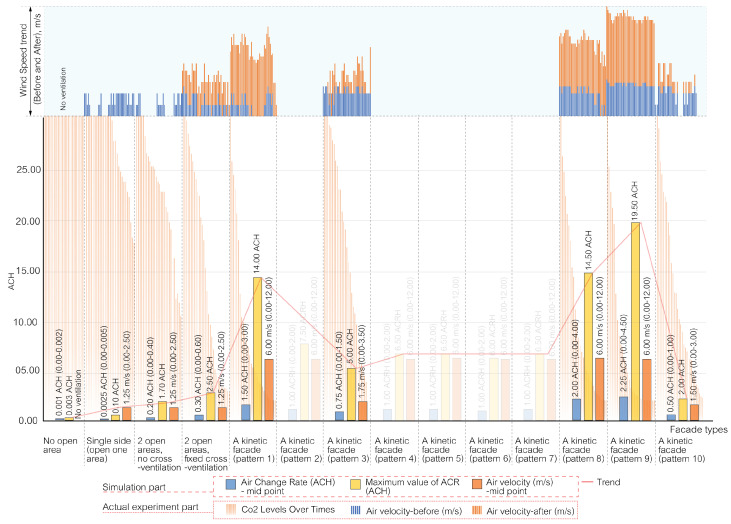
Comparative analysis of façade types: a synthesis of simulation and actual experiment results.

The comparative analysis, as illustrated in Figure 15, further reinforces these findings. The study’s comprehensive investigation, which combined computational simulations with physical tests, highlighted the superior ventilation capabilities of kinetic façades, especially patterns 1, 8, and 9. These patterns not only showed theoretical promise but also demonstrated remarkable practical utility in ventilating spaces. In contrast, static façades, particularly those with limited or single openings, showed significantly lower ventilation efficacy. This stark difference underscores the transformative potential of kinetic façades in architectural design.

The study also revealed the nuanced roles of different façade patterns. While patterns 1, 8, and 9 excelled in ventilation, patterns 3 and 10 carved out a niche in enhancing long-term human comfort, despite their lower effectiveness in air purification. This finding is crucial, as it highlights the importance of a multifaceted approach to façade design: one that considers both air quality and occupant comfort. The benefits of double-sided façades, particularly in terms of cross-ventilation efficiency, were also evident. However, patterns 2, 4, 5, 6, and 7 were less effective, primarily due to their limited impact on air exchange rates and velocities.

Contextually, the study aligns with the expanding research on dynamic, responsive building designs. The kinetic façades, inspired by the Mimosa plant, represent a notable advancement in this field, introducing a unique method for managing indoor air quality and human comfort. This is particularly relevant in the context of urban environments such as Bangkok, where the challenges of heat and humidity are persistent, thereby contributing to the discourse on sustainable building practices.

The implications of these findings are substantial for architectural design, especially in hot and humid climates. The demonstrated capability of kinetic façades in efficiently ventilating airborne particles while optimizing human comfort could significantly influence future building codes and standards, advocating for the integration of dynamic façade systems to enhance indoor environmental quality.

However, this study is not without limitations. Its focus on Bangkok’s specific climatic conditions may restrict the applicability of its findings to other climates. Moreover, the reliance on simulations and controlled experiments, while valuable, might not entirely reflect the complexities encountered in real-world building environments. These aspects highlight areas for further research and consideration in the application of kinetic façade systems.

## 7. Conclusions and Future Studies

The research findings suggest that kinetic façades have the potential to significantly improve indoor air quality and optimize human comfort. However, there is still work to be carried out in achieving a harmonious balance between these components.

**Figure 16:**
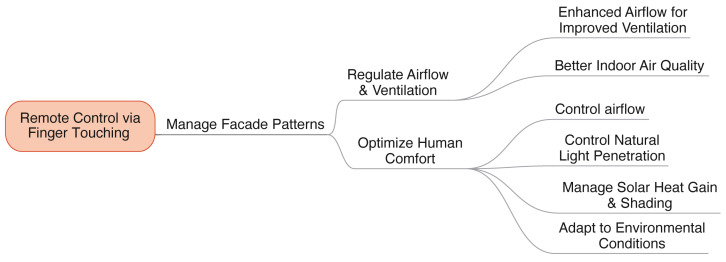
The guidelines for utilizing the Mimosa kinetic façade.

The research suggests that kinetic façades could play a crucial role in future architectural advancements by enhancing indoor air quality, improving human comfort, and increasing productivity. As depicted in Figure 16, maximizing this potential necessitates the integration of the most effective patterns into the kinetic façade design. One such pattern is the Mimosa kinetic façade, which is characterized by its touch-based adjustability. This innovative approach allows for the regulation of airflow and ventilation, leading to improved indoor environmental conditions. The design of the Mimosa kinetic façade also includes features such as the modulation of airflow, effective management of natural light, mitigation of solar heat gain, and provision of shading. Additionally, the façade’s design is adaptable to different climates, allowing users to modify patterns based on their comfort needs and environmental conditions.

This research identifies several key areas for future exploration in kinetic façades. These include studying their performance in various climates to assess adaptability and effectiveness, investigating long-term sustainability and practicality, and exploring seamless integration with smart technologies for enhanced efficiency and user experience. Additionally, researching other responsive organisms for innovative façade design insights, applying computational fluid dynamics and AI to improve façade movement predictions, and advancing material science and engineering fields to boost the façades’ adaptive capabilities are crucial. Assessing cost effectiveness and financial viability to promote broader adoption and evaluating practicality, economic feasibility, and maintenance in large-scale projects, focusing on airflow regulation and human comfort, are also essential. In summary, further research is vital for developing cost-effective, sustainable kinetic façades that meet environmental needs and enhance indoor human wellbeing.

## Figures and Tables

**Table 1:** The relationship between wind speed and human comfort.

Wind Speed Range (m/s)	Human Body Feeling
<1.0	Breezeless
1.0~5.0	Comfortable
5.0~10.0	Uncomfortable, movement affected
10.0~15.0	Very uncomfortable, movement greatly affected
15.0~20.0	Intolerable
>20.0	Dangerous

**Table 2:** Indoor air quality index.

IAQ Index	
CO${}_{2}$ (ppm)	Hazard Level
<700	Good
800	Moderate
1100	Poor
1500	Unhealthy
2000	Very Unhealthy
3000	Hazardous
>5000	Extreme

**Table 3:** Variables used in computational simulation (all façade types) and actual testing (*Mimosa pudica* façade concept) for assessing the potential of kinetic façades.

Simulation Variable	Actual Experiment Variable
1. Independent variable	1. Independent variable
1.1 Façade types	1.1 Façade patterns
1.2 Box size: 378 cm × 720 cm × 250 cm	
2. Dependent variable	2. Dependent variable
2.1 Air velocity: The speed of indoor air movement influencing human comfort and airborne contaminant reduction.	2.1 CO${}_{2\;}$ rate (in the setting area)
2.2 Air change rate (ACR): The rate of outdoor air replacement in a workspace, vital for indoor air quality and reducing health risks.	2. Air velocity (in the setting area)
	3. Potential for airborne particle ventilation (in the setting area)
3. Control variable	3. Control variable
3.1 The number of occupants	3.1 The level of airborne particles
3.2 External Conditions: This research considers the impact of outdoor factors such as temperature, humidity, and wind on the performance of kinetic façades, specifically in the context of Bangkok, Thailand’s weather conditions.	3.2 The actual experiment was conducted in a controlled environment in Bangkok, Thailand.
3.3 Building type: Office or workspaces used to assess the kinetic façade’s potential.	

**Table 4:** Summary of the measurement of air velocity with regard to human comfort (Table 1).

Façade Types	Air Velocity Range (m/s)	Airborne Ventilation	Human Comfort
No open area	no air ventilation	NO	NO
Single-sided façade (one open area)	0.00–2.50	NO	YES
Single-sided façade (two open areas, no cross ventilation)	0.00–2.50	NO	YES
Double-sided (two open areas, fixed cross ventilation)	0.00–3.50	NO	YES
Mimosa-inspired façade (pattern 1)	0.00–12.00	YES	NO
Mimosa-inspired façade (pattern 2)	0.00–12.00	YES	NO
Mimosa-inspired façade (pattern 3)	0.00–3.50	NO	YES
Mimosa-inspired façade (pattern 4)	0.00–12.00	YES	NO
Mimosa-inspired façade (pattern 5)	0.00–12.00	YES	NO
Mimosa-inspired façade (pattern 6)	0.00–12.00	YES	NO
Mimosa-inspired façade (pattern 7)	0.00–12.00	YES	NO
Mimosa-inspired façade (pattern 8)	0.00–12.00	YES	NO
Mimosa-inspired façade (pattern 9)	0.00–12.00	YES	NO
Mimosa-inspired façade (pattern 10)	0.00–3.00	NO	YES

**Table 5:** Rankings of façade types based on CO${}_{2}$ concentration levels at the conclusion of a 30 min evaluation period, as detailed in Table 2.

Façade Types	CO${}_{2}$ Concentration (ppm)	IAQI Rating	Interpretation
The kinetic façade (patterns 1, 8, and 9)	400	Good	This façade type exhibited the best performance, achieving the lowest CO${}_{2}$ concentration and securing a “Good” rating on the IAQI.
The kinetic façade (pattern 3)	440	Good	This façade type exhibited the best performance, achieving the lowest CO${}_{2}$ concentration and securing a “Good” rating on the IAQI.
The kinetic façade (pattern 10)	450	Good	This façade type had a marginally higher CO${}_{2\;}$ concentration than pattern 3 but remained within the “Good” IAQI rating.
Double-sided façade (two open areas with fixed cross ventilation)	550	Good	Despite its effective ventilation design, it recorded a higher CO${}_{2}$ concentration than the kinetic façades.
Single-sided façade (two open areas, no cross ventilation)	1410	Unhealthy	The absence of cross ventilation hindered its performance, resulting in an “Unhealthy” IAQI rating.
Single-sided façade (one open area)	3500	Hazardous	Its design, with only one open area, limited its ventilation potential, leading to a “Hazardous” IAQI rating.
Façade without open area	6000	Extreme	This façade type demonstrated the poorest performance, with the highest CO${}_{2}$ concentration, resulting in an “Extreme” IAQI rating.

## Data Availability

Data are contained within the article.

## References

[1] Awada M., Becerik-Gerber B., White E., Hoque S., O’Neill Z., Pedrielli G., Wen J., Wu T. (2022). Occupant health in buildings: Impact of the COVID-19 pandemic on the opinions of building professionals and implications on research. Build. Environ..

[2] Hagihara T., Toyota M. (2020). Mechanical Signaling in the Sensitive Plant *Mimosa pudica* L. Plants.

[3] Patil H.S., Vaijapurkar S. (2007). Study of the Geometry and Folding Pattern of Leaves of Mimosa pudica. J. Bionic Eng..

[4] Alonso M.J., Liu P., Marman S.F., J⌀rgensen R.B., Mathisen H.M. (2023). Holistic methodology to reduce energy use and improve indoor air quality for demand-controlled ventilation. Energy Build..

[5] Wang H., Li X., Zhang Y., Hopke P.K., Mandin C. (2022). Visualization and Measurement of Indoor Airflow by Color Sequence Enhanced Particle Streak Velocimetry. Handbook of Indoor Air Quality.

[6] Gosztonyi S. (2018). The role of geometry for adaptability: Comparison of shading systems and biological role models. J. Facade Des. Eng..

[7] Hosseini S.M., Mohammadi M., Rosemann A., Schröder T., Lichtenberg J. (2019). A morphological approach for kinetic façade design process to improve visual and thermal comfort: Review. Build. Environ..

[8] Attia S., Navarro A.L., Juaristi M., Monge-Barrio A., Gosztonyi S., Al-Doughmi Z. (2018). Post-occupancy evaluation for adaptive façades. J. Facade Des. Eng..

[9] Jamrozik A., Clements N., Hasan S.S., Zhao J., Zhang R., Campanella C., Loftness V., Porter P., Ly S., Wang S. (2019). Access to daylight and view in an office improves cognitive performance and satisfaction and reduces eyestrain: A controlled crossover study. Build. Environ..

[10] Saelens D., Carmeliet J., Hens H. (2011). Energy Performance Assessment of Multiple-Skin Facades. HVAC R Res..

[11] Attia S., Favoino F., Loonen R.C.G.M., Petrovski A., Monge-Barrio A. (2015). Adaptive façades system assessment: An initial review. Proceedings of the 10th Conference on Advanced Building Skins.

[12] Struck C., Almeida M., Silva S., Mateus R., Lemarchand P., Petrovski A., Rabenseifer R., Wansdronk R., Wellershoff F., Wit J. Adaptive facade systems—Review of performance requirements, design approaches, use cases and market needs. Proceedings of the 10th Energy Forum on Advanced Building Skins.

[13] Attia S., Bilir S., Safy T., Struck C., Loonen R., Goia F. (2018). Current trends and future challenges in the performance assessment of adaptive façade systems. Energy Build..

[14] Bacha C.B., Bourbia F. Effect of kinetic facades on energy efficiency in office buildings -hot dry climates. Proceedings of the 11th Conference on Advanced Building Skins.

[15] Lee E.S., Selkowitz S.E., DiBartolomeo D.L., Klems J.H., Clear R.D., Konis K.S., Hitchcock R., Yazdanian Y., Mitchell R., Konstantoglou M. (2009). High Performance Building Facade Solutions: PIER Final Project Report. California Energy Commission Public Interest Energy Research Program. https://eta-publications.lbl.gov/sites/default/files/lbnl-4583e.pdf.

[16] Romano R., Aelenei L., Aelenei D., Mazzucchelli E.S. (2018). What is an adaptive façade? Analysis of recent terms and definitions from an international perspective. J. Facade Des. Eng..

[17] Prieto A., Knaack U., Klein T., Auer T. (2018). Possibilities and constraints for the widespread application of solar cooling integrated façades. J. Facade Des. Eng..

[18] Basarir B., Altun M.C. (2018). A redesign procedure to manufacture adaptive façades with standard products. J. Facade Des. Eng..

[19] Casini M. (2018). Active dynamic windows for buildings: A review. Renew. Energy.

[20] Bakker L.G., van Oeffelen E.C.M.H., Loonen R.C.G.M., Hensen J.L.M. (2014). User satisfaction and interaction with automated dynamic facades: A pilot study. Build. Environ..

[21] de Klijn-Chevalerias M.L., Loonen R.C., Zarzycka A., de Witte D., Sarakinioti M.V., Hensen J.L. (2017). Assisting the development of innovative responsive façade elements using building performance simulation. Simul. Ser..

[22] Elzeyadi I. (2017). The impacts of dynamic façade shading typologies on building energy performance and occupant’s multi-comfort. Archit. Sci. Rev..

[23] Paper C., Pierleoni A., Spa F., Politecnico V.S., Politecnico L.B. Innovative technologies for transparent building envelopes: Experimental assessment of energy and thermal comfort data to facilitate the decision-making process. Proceedings of the CIGOS Innovatiion and Construction, CIGOS Innovatiion and Construction.

[24] Loonen R.C.G.M., Hoes P., Hensen J.L.M. Performance prediction of buildings with responsive building elements challenges and solutions. Proceedings of the 2014 Building Simulation and Optimization Conference (BSO14).

[25] Chang T.W., Huang H.Y., Datta S. (2019). Design and fabrication of a responsive carrier component envelope. Buildings.

[26] Saelens D., Hens H. Evaluating the Thermal Performance of active envelopes. Proceedings of the Buildings VIII: Thermal Performance of Exterior Envelopes of Whole Buildings.

[27] Jayathissa P., Caranovic S., Hofer J., Nagy Z., Schlueter A. (2018). Performative design environment for kinetic photovoltaic architecture. Autom. Constr..

[28] Loonen R.C.G.M., Trčka M., Cóstola D., Hensen J.L.M. (2013). Climate adaptive building shells: State-of-the-art and future challenges. Renew. Sustain. Energy Rev..

[29] Chayaamor-Heil N., Vitalis L. (2021). Biology and architecture: An ongoing hybridization of scientific knowledge and design practice by six architectural offices in France. Front. Archit. Res..

[30] Li C., Tang H. (2021). Study on ventilation rates and assessment of infection risks of COVID-19 in an outpatient building. J. Build. Eng..

[31] Srivastava S., Zhao X., Manay A., Chen Q. (2021). Effective ventilation and air disinfection system for reducing coronavirus disease 2019 (COVID-19) infection risk in office buildings. Sustain. Cities Soc..

[32] Pourshab N., Tehrani M., Toghraie D., Rostami S. (2020). Application of double glazed façades with horizontal and vertical louvers to increase natural air flow in office buildings. Energy.

[33] Dao H.T., Kim K.S. (2022). Behavior of cough droplets emitted from Covid-19 patient in hospital isolation room with different ventilation configurations. Build. Environ..

[34] Hurraß J., Golmohammadi R., Bujok S., Bork M., Thelen F., Wagner P., Exner D., Schönfeld C., Hornei B., Kampf G. (2022). Explosive COVID-19 outbreak in a German nursing home and the possible role of the air ventilation system. J. Hosp. Infect..

[35] Blocken B., van Druenen T., Ricci A., Kang L., van Hooff T., Qin P., Xia L., Ruiz C.A., Arts J.H., Diepens J.F. (2021). Ventilation and air cleaning to limit aerosol particle concentrations in a gym during the COVID-19 pandemic. Build. Environ..

[36] Bergefurt L., Weijs-Perrée M., Appel-Meulenbroek R., Arentze T. (2022). The physical office workplace as a resource for mental health—A systematic scoping review. Build. Environ..

[37] Harweg T., Bachmann D., Weichert F. (2021). Agent-based simulation of pedestrian dynamics for exposure time estimation in epidemic risk assessment. J. Public Health.

[38] Elsaid A.M., Ahmed M.S. (2021). Indoor Air Quality Strategies for Air-Conditioning and Ventilation Systems with the Spread of the Global Coronavirus (COVID-19) Epidemic: Improvements and Recommendations. Environ. Res..

[39] Agarwal N., Meena C.S., Raj B.P., Saini L., Kumar A., Gopalakrishnan N., Kumar A., Balam N.B., Alam T., Kapoor N.R. (2021). Indoor air quality improvement in COVID-19 pandemic: Review. Sustain. Cities Soc..

[40] Martins N.R., da Graça G.C. (2018). Effects of airborne fine particle pollution on the usability of natural ventilation in office buildings in three megacities in Asia. Renew. Energy.

[41] Fordham M. (2000). Natural ventilation. Renew. Energy.

[42] Aflaki A., Mahyuddin N., Mahmoud Z.A.C., Baharum M.R. (2015). A review on natural ventilation applications through building façade components and ventilation openings in tropical climates. Energy Build..

[43] Sankaewthong S., Horanont T., Miyata K., Karnjana J., Busayarat C., Xie H. (2022). Using a Biomimicry Approach in the Design of a Kinetic Façade to Regulate the Amount of Daylight Entering a Working Space. Buildings.

[44] Holzer P., Psomas T. (2018). Ventilative Cooling Sourcebook: Energy in Buildings and Communities Programme. Department of Civil Engineering, Aalborg University, Thomas Manns Vej 23, 9220, Aalborg Ø, Denmark. EBC Bookshop. https://www.aivc.org/resource/ventilative-cooling-source-book.

[45] Su X., Yuan Y., Wang Z., Liu W., Lan L., Lian Z. (2023). Human thermal comfort in non-uniform thermal environments: A review. Energy Built Environ..

[46] Hou Y. (2018). Effect of wind speed on human thermal sensation and thermal comfort. AIP Conf. Proc..

[47] Olesen B.W. (2004). International standards for the indoor environment. Indoor Air Suppl..

[48] Maddalena R., Mendell M.J., Eliseeva K., Chan W.R., Sullivan D.P., Russell M., Satish U., Fisk W.J. (2015). Effects of ventilation rate per person and per floor area on perceived air quality, sick building syndrome symptoms, and decision-making. Indoor Air.

[49] Persily A.K. (2000). Rationale for Ventilation Rate Requirements in ASHRAE Standard 62-1999 and in Potential Revisions to the Standard. Air Quality and Comfort in Airliner Cabins.

[50] Mendell M.J., Apte M.G. (2010). Balancing Energy Conservation and Occupant Needs in Ventilation Rate Standards for “Big Box” Stores and Other Commercial Buildings in California: Issues Related to the ASHRAE 62.1 Indoor Air Quality Procedure.

[51] Speck O., Speck D., Horn R., Gantner J., Sedlbauer K.P. (2017). Biomimetic bio-inspired biomorph sustainable? An attempt to classify and clarify biology-derived technical developments. Bioinspir. Biomim..

[52] Silverstein D., Samuel P., Decarlo N. (2011). Biomimicry. The Innovator’s Toolkit.

[53] Hapsari F.N., Purwaningsih R., Azzahra F., Sari D.P. (2022). Velcro Product Design with Biomimicry Approaches. IOP Conf. Ser. Earth Environ. Sci..

[54] Visser W., Benyus J.M., Visser W. (2013). Biomimicry. The Top 50 Sustainability Books.

